# Association mapping of drought tolerance and agronomic traits in rice (*Oryza sativa* L.) landraces

**DOI:** 10.1186/s12870-021-03272-3

**Published:** 2021-10-23

**Authors:** Radha Beena, Silvas Kirubakaran, Narayanan Nithya, Alagu Manickavelu, Rameshwar Prasad Sah, Puthenpeedikal Salim Abida, Janardanan Sreekumar, Poolakkal Muhammed Jaslam, Rajendrakumar Rejeth, Vijayalayam Gengamma Jayalekshmy, Stephen Roy, Ramakrishnan Vimala Manju, Mariasoosai Mary Viji, Kadambot H. M. Siddique

**Affiliations:** 1 Department of Plant Physiology, College of Agriculture, Vellayani, Kerala Agricultural University, Thiruvananthapuram, Kerala India; 2 BASF, Morrisville, NC 27560 USA; 3 Department of Genomic Science, Central University of Kerala, Kasaragod, Kerala India; 4 Indian Council of Agricultural Research (ICAR)-Central Rice Research Institute, currently named National Rice Research Institute (NRRI), Cuttack, Odisha India; 5 Regional Agricultural Research Station, Pattambi, Kerala Agricultural University, Palakkad, Kerala India; 6 Indian Council of Agricultural Research (ICAR)-Central Tuber Crops Research Institute, Sreekaryam, Thiruvananthapuram, Kerala India; 7 Chaudhary Charan Singh (CCS) Haryana Agricultural University, Hissar, India; 8 Department of Plant Breeding and Genetics, College of Agriculture, Vellayani, Kerala Agricultural University, Thiruvananthapuram, Kerala India; 9 The UWA Institute of Agriculture, The University of Western Australia, Perth, WA Australia

## Abstract

**Background:**

Asian cultivars were predominantly represented in global rice panel selected for sequencing and to identify novel alleles for drought tolerance. Diverse genetic resources adapted to Indian subcontinent were not represented much in spite harboring useful alleles that could improve agronomic traits, stress resilience and productivity. These rice accessions are valuable genetic resource in developing rice varieties suited to different rice ecosystem that experiences varying drought stress level, and at different crop stages. A core collection of rice germplasm adapted to Southwestern Indian peninsular genotyped using SSR markers and characterized by contrasting water regimes to associate genomic regions for physiological, root traits and yield related traits. Genotyping-By-Sequencing of selected accessions within the diverse panel revealed haplotype variation in genic content within genomic regions mapped for physiological, morphological and root traits.

**Results:**

Diverse rice panel (99 accessions) were evaluated in field and measurements on plant physiological, root traits and yield related traits were made over five different seasons experiencing varying drought stress intensity at different crop stages. Traits like chlorophyll stability index, leaf rolling, days to 50% flowering, chlorophyll content, root volume and root biomass were identified as best predictors of grain yield under stress. Association mapping revealed genetic variation among accessions and revealed 14 genomic targets associated with different physiological, root and plant production traits. Certain accessions were found to have beneficial allele to improve traits, plant height, root length and spikelet fertility, that contribute to the grain yield under stress. Genomic characterization of eleven accessions revealed haplotype variation within key genomic targets on chromosomes 1, 4, 6 and 11 for potential use as molecular markers to combine drought avoidance and tolerance traits. Genes mined within the genomic QTL intervals identified were prioritized based on tissue specific expression level in publicly available rice transcriptome data.

**Conclusion:**

The genetic and genomic resources identified will enable combining traits with agronomic value to optimize yield under stress and hasten trait introgression into elite cultivars. Alleles associated with plant height, specific leaf area, root length from PTB8 and spikelet fertility and grain weight from PTB26 can be harnessed in future rice breeding program.

**Supplementary Information:**

The online version contains supplementary material available at 10.1186/s12870-021-03272-3.

## Background

Globally, rice (*Oryza sativa* L.) is a staple food grown in 144 million hectares span across 114 countries [1] to meet population explosion [2]. Global rice production was revolutionized after first green revolution with production increment of 140% by doubling of average productivity from 2.23 to 4.32 t/ha [3]. However, increased production of about 60% is expected to meet future global needs [4]. Indian rice production is estimated to be 148.26 million metric tons from land area of 44.1 million hectares, in which one-half area is irrigated [5]. Drought stress is one of the major threat to the rice production in the Asian-Pacific region, affects yield stability in rainfed ecosystems [6]. Development of drought-resistant rice cultivars is critical to reduce climate-related risk, maintain productivity, and enhance livelihood of rice growers [7].

Drought tolerance consists of three major components involving interaction among physiological, morphological, biochemical traits [8]. The dehydration avoidance related traits maintain plant water status through enhanced root water uptake and reduced water loss in leaf through regulation of leaf area, stomatal conductance, canopy temperature and photosynthetic rate to result in high yield potential under water limitation. Leaf morphological, phenological, physiological and biochemical traits and their modulation is critical in maintaining plant water use and protect yield under water stress is well established in crop plants [9–17]. Roots are the first organ to experience water stress [18] and strongly associated with grain yield under stress [15, 19]. Later, root traits were viewed as target traits to improve drought adaptation [20, 21] and regulate the canopy temperature through stomatal regulation [22]. The increase in yielding potential was observed by introgression of root trait [23]. Number of studies have been done to establish the relevance of root traits for water uptake [24–29] and their importance in yield protection under stress environments [22, 23, 30]. Efforts have been made to identify genomic regions associated with root traits in major cereal crops like rice [31], wheat [32] and legumes [33–36]. Drought adaptation and biomass productivity under stress depends on the plant Water Use Efficiency (WUE) or instantaneous WUE [37–39].

The success in developing drought tolerant germplasm achieved so far is attributed through choice of parents, selection criteria, and robustness of the managed screening protocols [40]. The future genetic improvements in rice productivity will be achieved by adapting holistic approach that integrates plant breeding with physiological dissection of resistance traits and molecular genetic/genomic tools together with agronomical management practices. Alternative to conventional mapping approach, selective genotyping was successfully utilized in rice breeding to map major QTLs for secondary traits [10, 13, 24, 41] and grain yield under stress [42]. Most of these Quantitative Trait Loci (QTLs) identified using bi-parental or multi-parental populations have limited the availability of allelic diversity and reduced genomic resolution for positional cloning process [43–46]. Genome Wide Association Study (GWAS) has emerged as a powerful approach to address limitations in traditional mapping approach and identify genes governing complex traits like rice root [47–52] and production traits [45, 53–55]. This approach has been successfully used to pinpoint root trait related genes associated with taproot cell length [56] and cortex cell properties [30].

To meet the global food demand, rich genetic diversity within both domestic and rice wild relatives should be explored towards genetic improvement of rice cultivars [57–59]. Brozynska et al. [60] reviewed the importance of crop wild relatives as a contributor of novel alleles to improve food security and stress adaptation [33, 34, 61–63]. Genome comparison among 3010 diverse accessions from Asia [64] exhibited huge divergence for novel alleles towards crop improvement. Local landraces and accessions from India are not widely represent in global rice panels [65, 66] in spite of useful genetic variation observed for various traits [10, 12, 13, 23–25]. The rice cultivars/accessions adapted to Southern India needs to be explored to identify novel genetic and genomic targets to improve stress resilience as Tamil Nadu and Kerala, are key rice target environments, highly affected by water limitation [12, 14, 23, 26]. The present study is conducted with the objectives, (1) Screen diverse rice accession adapted to target environment drought stress adaptive plant physiology, root and water use efficiency traits (2) Estimate genetic diversity, population structure and identify marker-trait association and (3) Select parental genotypes with better phenotypic values towards developing rice cultivars with drought tolerance and productivity in water scarce environments.

## Results

### Phenotypic variation for morpho-physiological and plant production traits in panel I

Diverse rice accession within Panel I (Supplementary Table 1) exhibited significant variation for morpho-physiological and plant production traits under water stress condition in trials 1 and 2 (Tables 1 and 2). The amount of precipitation and relative humidity observed in the trial sites are provided in Supplementary Figure 1. The effect of water stress is evident in trial II with higher percent change in leaf temperature (6 °C) than trial 1 with 3 °C resulted in reduction of plant height in trial 2 (5.35%), in contrast to trial 1 with an average reduction of 4.58%. Similar effect of water stress was observed for other production traits like days to 50% flowering, tiller number, yield per plant, and 1000-grain weight (Tables 1 and 2). Irrespective of the trials 1 and 2 under water stress treatment, PTB 7 produced higher tiller number with a range of 5 to 11. The PTB55 and Prathyasha recorded the least and higher leaf rolling scores of 1.78 and 7.78, respectively. In dry season of trial 2, the PTB 27 was found to maintain higher plant water status, which is evident with higher relative water content of 73%, than elite variety ADT37. PTB 35 recorded highest phenotypic values for yield per plant, 1000-grain weight through maintaining higher percent spikelet fertility traits in both trials. The relative yield reduction between rainfed and irrigated condition was lower in two accessions, Gopika and Shreyas with a reduction of 15 and 18%, respectively. These accessions also recorded lower drought susceptibility index of 1.14 and 1.17, whereas PTB25 exhibited lower DSI across trials. The association between grain yield, physiological and plant production traits in trial 1 and 2 are detailed in Supplementary Tables 2 and 3.

**Table 1:** Descriptive analysis of phenotypic, physiological and yield traits measured in Trial 1

Traits	Mean		Range		Percentage change	***P***-value
	WS	C	WS	C		
Plant height (cm)	110.74 ± 12.71	116.05 ± 12.78	82.50–135.00	87.50–144.10	−4.58	0.000251774
Tiller number	7.53 ± 1.22	10.25 ± 1.39	5.00–11.00	7.00–14.00	−26.54	2.04E-41
Days to 50% flowering	94.65 ± 10.01	88.47 ± 10.81	67.50–119.00	61.50–114	6.99	3.8E-07
Leaf temperature (°C)	28.26 ± 0.589	27.50 ± 0.533	26.95–29.00	26.00–29.35	2.76	3.16E-27
Leaf rolling score	4.80 ± 1.38	–	1.78–7.78	–	–	
Leaf drying score	1.93	–	1.56–9.00	–	–	
Relative water content (%)	65.19 ± 4.78	84.13 ± 3.22	57.34–78.72	75.70–90.17	−22.51	1.3E-127
Cell membrane stability index (%)	84.95 ± 3.72	–	80.28–94.36	–	–	–
Chlorophyll stability index (%)	86.14 ± 3.57	–	80.36–95.59	–	–	–
Yield/plant (g)	9.55 ± 1.89	12.94 ± 2.38	6.55–14.47	8.33–19.64	−26.20	1.05E-34
Thousand grain weight (g)	22.71 ± 1.48	23.55 ± 1.41	17.55–25.35	18.70–25.95	−3.57	5.71E-07
Spikelet fertility percentage (%)	65.26 ± 4.58	75.43 ± 4.75	54.19–78.22	64.96–86.91	−13.48	2E-55
Percentage relative yield reduction	25.48 ± 4.64	–	14.84–43.81	–	–	–
Drought susceptibility index	1.00 ± 0.062	–	0.86–1.14	–	–	–
Soil moisture (%)	–	–	12.4–30.96	–	–	–

*WS* water-stressed, *C* Irrigated condition

**Table 2:** Descriptive analysis of phenotypic, physiological and yield traits measured in Trial 2

Traits	Mean		Range		Percentage change	***P***-value
	WS	C	WS	C		
Plant height (cm)	106.28 ± 12.56	112.29 ± 12.28	80.15–130	86.50–140.50	−5.35	2.12E-05
Tiller number	6.34 ± 0.845	8.88 ± 1.23	5.00–8.50	6.50–13.00	−28.60	7.87E-52
Days to 50% flowering	91.26 ± 10.86	85.78 ± 10.36	65.50–115.50	63.00–112.00	6.38	6.58E-06
Leaf temperature (°C)	29.56 ± 0.548	27.94 ± 0.385	27.30–30.55	27.10–28.70	5.79	6.78E-25
Leaf rolling score	4.05 ± 1.39	–	1.11–7.84	–	–	–
Leaf drying score	4.00 ± 1.37	–	1.17–7.67	–	–	–
Relative water content (%)	61.07 ± 4.79	78.89 ± 3.89	50.35–72.98	70.36–86.26	−22.58	7.4E-114
Cell membrane stability index (%)	81.83 ± 3.70	–	75.53–92.24	–	–	–
Chlorophyll stability index (%)	82.11 ± 3.85	–	75.52–92.85	–	–	–
Yield/plant (g)	8.40 ± 1.57	11.55 ± 2.15	6.06–12.17	7.94–18.62	−27.27	1.81E-37
Thousand grain weight (g)	21.78 ± 1.33	22.66 ± 1.35	17.15–24.00	17.45–24.95	−3.88	3.16E-08
Spikelet fertility percentage (%)	61.04 ± 4.39	70.91 ± 4.58	50.47–73.79	61.12–81.93	−13.91	1.24E-55
Percentage relative yield reduction	29.46 ± 4.96	–	17.91–46.94	–	–	–
Drought susceptibility index	1.00 ± 0.070	–	0.75–1.17	–	–	–
Soil moisture (%)	–	–	10.75–24.96	–	–	–

*WS* water-stressed, *C* Irrigated condition

### Phenotypic diversity for root traits and water use in panel II

The rainfed treatment of trial 3 and 4 received a total precipitation of 61 and 75 mm, respectively spanning a growth period of June to September (Supplementary Figure 1). In spite of lower precipitation in trial 3, the diverse rice panel II exhibited higher average phenotypic value for PH (110.4 cm), shoot biomass (19.3 g) and root biomass (12.6 g). Whereas in trial 4 recorded average phenotypic value for PH (108 cm), shoot biomass (18 g) and root biomass (5.5 g) (Table 3). To account for variation in the agro climatic condition across years (Trial 3 and 4), average phenotypic values across trials were calculated. In trials 3–4, lower CID values were observed in accessions, PTB7, PTB4, PTB8 and Kalladiyar, lower SLA [PTB26, 8, 54, 10, Kalladiyar, Chomala], average higher PH [PTB2, 10, 6, PTB 24, Thottacheera, Jeerakasala], Higher RL and RV [PTB1,2, 8, 10, 15, Chuvanna Modan, Kalladiyar], higher RB [PTB2, 10], and shoot biomass [PTB2, 10, 29), 26 and Kalladiyar].

**Table 3:** Descriptive statistics of plant morphological, physiological and root traits measured in diverse panel II in trials 3 and 4

Traits	Year	Min	Maximum	Mean	SD
Plant Height (cm)	**2011**	62.90	174.40	110.36	24.67
	**2013**	77.00	146.50	107.90	20.62
	**Average**	74.84	146.75	108.93	17.78
SPAD-Chlorophyll content (%)	**2011**	26.66	44.88	36.72	3.72
	**2013**	27.20	40.60	34.45	3.19
	**Average**	29.22	41.03	35.62	2.50
Shoot Biomass (g)	**2011**	4.08	29.24	19.26	7.32
	**2013**	7.20	31.81	17.95	5.78
	**Average**	8.81	28.77	18.60	4.75
Root Length (cm)	**2011**	27.40	90.60	40.97	10.56
	**2013**	35.60	96.00	51.61	11.59
	**Average**	33.20	72.80	46.18	7.71
Root Volume (cm^3^)	**2011**	8.40	74.00	31.25	13.96
	**2013**	13.30	90.00	37.40	18.73
	**Average**	14.50	78.00	34.20	12.54
Root Biomass (g)	**2011**	4.08	29.24	12.56	6.27
	**2013**	1.70	13.83	5.54	2.49
	**Average**	4.08	18.19	9.04	3.25

### Relatedness among phenological, physiological and yield traits under stress

**Fig. 1:**
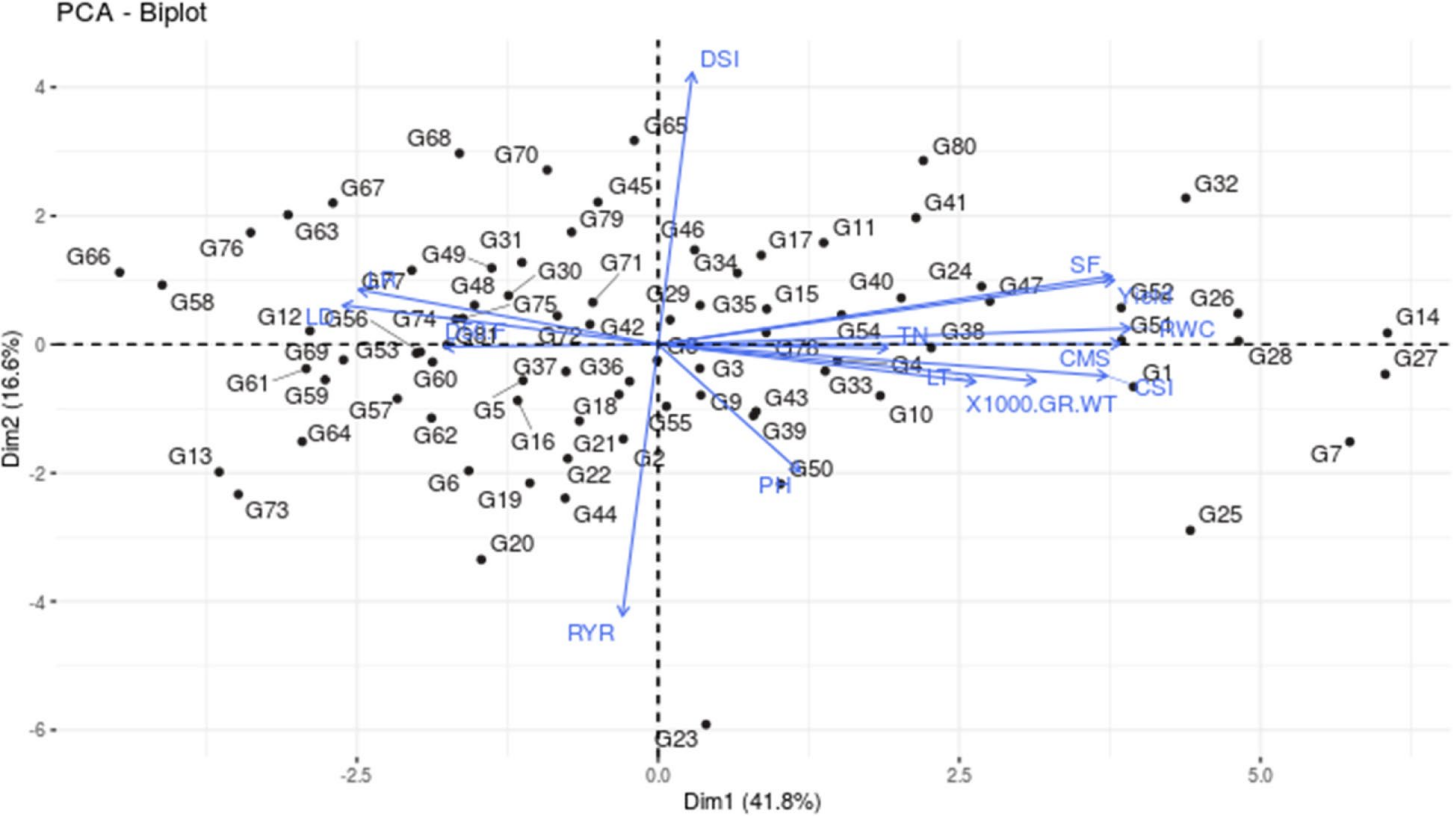
Genotype-by-trait-biplot analysis of 81 rice genotypes for two principal components under water stress. The encircled area depicted drought tolerant genotypes (G7-Parambuvattan (Ptb7); G14-Kavunginpoothala (Ptb15); G26-Kattamodan (Ptb28); G27-KaruthaModan (Ptb29); G28-ChuvannaModan (Ptb30)

In diverse panel I, the Principal Component Analysis (PCA) was performed for 14 morpho-physiological traits measured under water stress and well-watered conditions (Fig. 1). In Trial 1 water stress, the PCA revealed two principal components with Eigen values > 2 capturing 58.3% of the total variation. The plots for the Principal Component 1(PC1) vs. PC2 represented a higher phenotypic variation of 41.8, and 14.6%. PC1 was greatly influenced by traits relative water content, cell membrane stability index, chlorophyll stability index, yield, spikelet fertility percentage, drought susceptibility index, leaf rolling and leaf drying. In Trial 2, 2018 water stress, the PCA revealed two principal components with Eigen values > 1 capturing 52.7% of the total variation. The plots for the Principal Component 1 (PC1) vs. PC2 represented a higher phenotypic variation of 35.8%, and 16.9. In Trial 2, in addition to traits in Trial 1, plant height, tiller number, 1000-grain weight and relative yield ratio influenced PC1. Combining the data from trials 1 and 2, revealed two principal components with Eigen values > 3 capturing 50.6% of the total variation. The plots for the Principal Component 1 (PC1) vs. PC2 represented a higher phenotypic variation of 39.3, and 11.3%. PC1 was greatly influenced by traits days to 50 % flowering, drought susceptibility index, leaf temperature, spikelet fertility, relative water content and yield. Accessions PTB1, 4, 12, 15, 17, 19, 26, 28, 34, 35, 37, 40, 52, 55, 60, Uma were found to have higher grain yield and spikelet fertility. The accessions, PTB 2,3,5,32,33,38,39,46,51,56,57, N-22, Sampada, Jeerakashala, Bhadra, Makom and Prathyasha were found to have alleles to improve DFF and DSI in rainfed condition.

To understand the relationship between significant phenological, physiological traits, root traits and shoot biomass measured in field trials 3 and 4, PCA was performed. The PCA revealed three principal components with Eigen values > 1 capturing 60.6% of the total variation. The plots for the Principal Component 1(PC1) vs. PC2 represented a higher phenotypic variation (44.6%), with 27.2 and 17.4%, respectively. In 2011 study, PC1 was greatly influenced by traits SLA, RB, PH and SB. In 2013 study the PCA revealed three principal components with Eigen values > 1 capturing 65.5% of the total variation. The plots for the Principal Component 1(PC1) vs. PC2 represented a higher phenotypic variation (52.0%), with 35.6 and 16.4%, respectively. Under both experimental conditions, the PC1 was greatly influenced by PH and SB whereas the PC2 is influenced by physiological and root traits. Correlation among average phenotypic values across years on PH, SDW, RV, RL and RDW revealed plant height to be significantly associated with shoot biomass, root volume, and root biomass (Supplementary Table 4). The root traits, RL and RV are associated with both shoot and root biomass. The physiological traits, canopy temperature is positively correlated to plant height, negatively correlated to SPAD chlorophyll content and carbon isotope discrimination values measured.

### Predictors of grain yield in different drought stress intensity

Average data of 63 rice common accessions between trials 1 to 4 were used to identify traits that are good predictors of yield (Trial 1 and 2) and shoot biomass (Trials 3 and 4). The RWC, 1000GW, SF, CMS, CSI are determined to be best predictors of average grain yield in Trial 1 and 2. Shoot biomass is directly associated with grain yield under rainfed rice ecosystems. The average data on RB, RV, SPAD (trials 3 and 4), LT and RYR (Trials 1 and 2) were best predictors of shoot biomass measured. To identify the grain yield predictors under different intensity of drought stress imposed on trials 1, 2 and 5, average data on 35 accessions common across trials were analyzed. This analysis revealed CSI (Trials 1 and 2), LR (trial 5), DFF (Trials 1 and 2), Chlorophyll content (Trial 2), RV (Trials 3 and 4) and RB (Trials 3 and 4) were found to best predictors of grain yield under water stress condition in trial 5. Clustering of the 35 diverse accessions across trials 1–5 based on average RV and RB revealed 4 different clusters with four rice accessions [PTB1, 2,10 and 13] with higher average phenotypic value for RV and RB. Within this cluster, the accessions, PTB1,2, and 10 was found to be high yielding in trial 1–2, 4 and 5, also possess valuable alleles for root architectural traits. The average yield of PTB1 is 12 g, whereas N22 rice cultivar is only 7.2 g in Trials 1–2, whereas in trial 5, the average shoot biomass of landrace, Ponnaryan (PTB2) across years is 27.6 g, which is on par to rice hybrid, PHB71 (28.6 g), and better than N22, a known rice cultivar to be drought and heat tolerant with 20.3 g.

### Genotypic relatedness of diverse accession panels

**Fig. 2:**
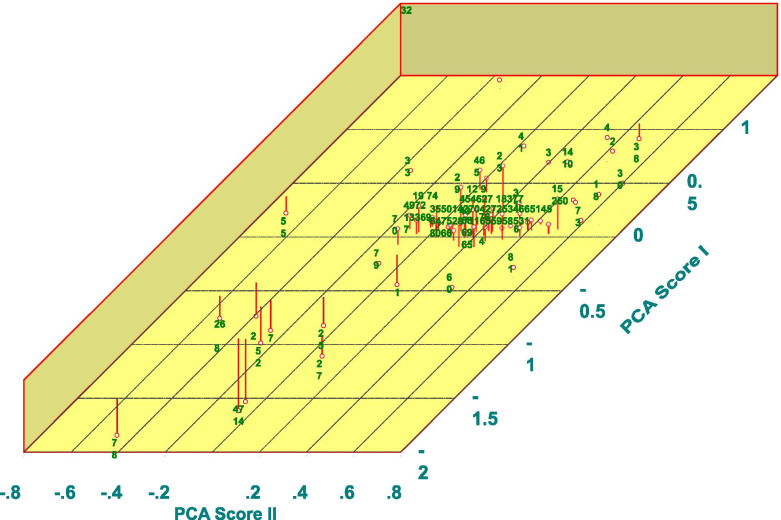
Three dimensional of Principal Co-ordinate analysis of 81 rice diverse panel II genotyped with 100 Simple Sequence Repeat markers

**Fig. 3:**
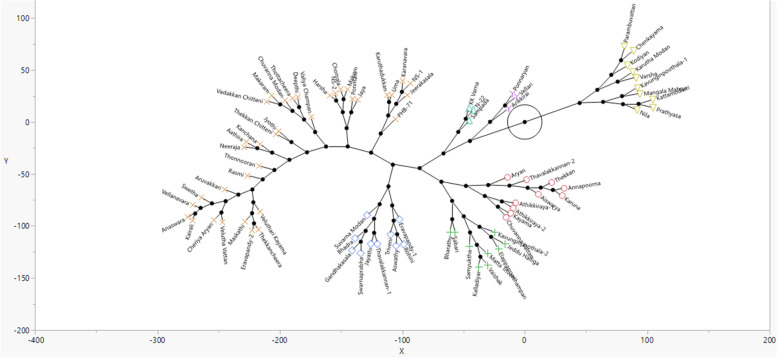
Constellation plot to show genetic relatedness among 81 diverse rice of Panel II based on hierarchial clustering analysis

Genetic related analysis of Panel I revealed nine different clusters, including seven clusters only with local rice landraces harbor valuable alleles to improve different agronomic traits in rice lines adapted to rainfed ecosystem. Interestingly, Aryan (PTB1) and PTB10 (Thekkancheera) are genetically closer, whereas accession, PTB2 (Ponnaryan) is genetically dissimilar to any high yielding cultivars evaluated in this study. To illustrate the genetic relatedness among diverse rice lines of Panel II, Principal coordinate analysis (PCoA) was performed (Fig. 2) reveled four quadrants with 56, 4, 8 and 13 genotypes, respectively. The fourth quadrant is a mixture with a pool of rice accessions with known drought tolerance [PTB30 (ChuvannaModan)], and other agronomic traits [PTB 20, 33, 34, 47, 56, Gandhakasala, Sampada]. To gain more insights on the diversity and relatedness among diverse accessions, a hierarchical clustering was performed to derive a phylogenetic analysis representing seven clusters. Further mining of diverse accessions within in fourth quadrant, revealed PTB30 (Chuvanna Modan), PTB20 (Vadakkan Chitteni), PTB47 (Neeraja), PTB34 (Valiya Champan) are genetically similar than other accessions, Gandhakasala, PTB56 (Varsha), PTB33 (Arikkirai), and Sampada. PCA of marker data revealed a cluster with accession Ponnaryan with higher yield under stress and root architectural traits, is genetically similar to Vellari (Fig. 3).

### Population structure and linkage disequilibrium analysis

**Fig. 4:**
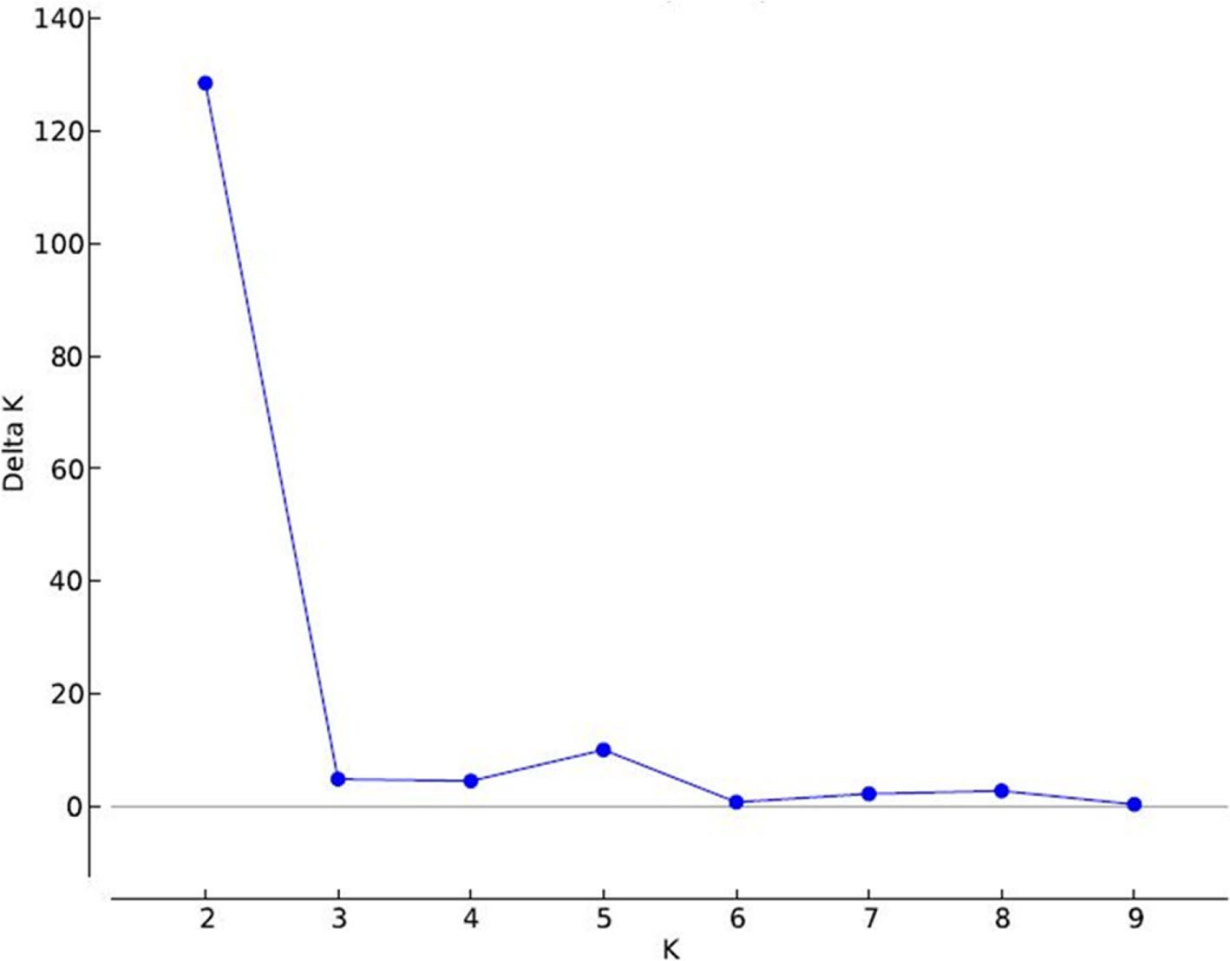
Estimates of subpopulations analysis of 81 diverse rice panels revealed 2 sub population using delta K-values with STRUCTURE program

Population structure analysis among 81 rice genotypes with a set of 100 SSR markers determined optimum K value of two, representing the diverse panel group into two major clusters (Fig. 4). In Panel I, first cluster comprised 65 genotypes and the second cluster with 16 genotypes including 6 admixture accessions. In Panel II, the first cluster comprised known to include drought susceptible genotypes but high yielding genotypes suited to irrigated condition, whereas the second cluster comprised known drought tolerant genotype, Nagina-22. Certain genotypes in cluster 2 had higher average phenotypic values across years than known drought tolerant genotype Nagina-22 for PH and SPAD [PTB28], RL [PTB1, 34 and Sabari), RV (genotypes in cluster 2), RB (PTB1) and SB (PTB60-Vaishak).

**Fig. 5:**
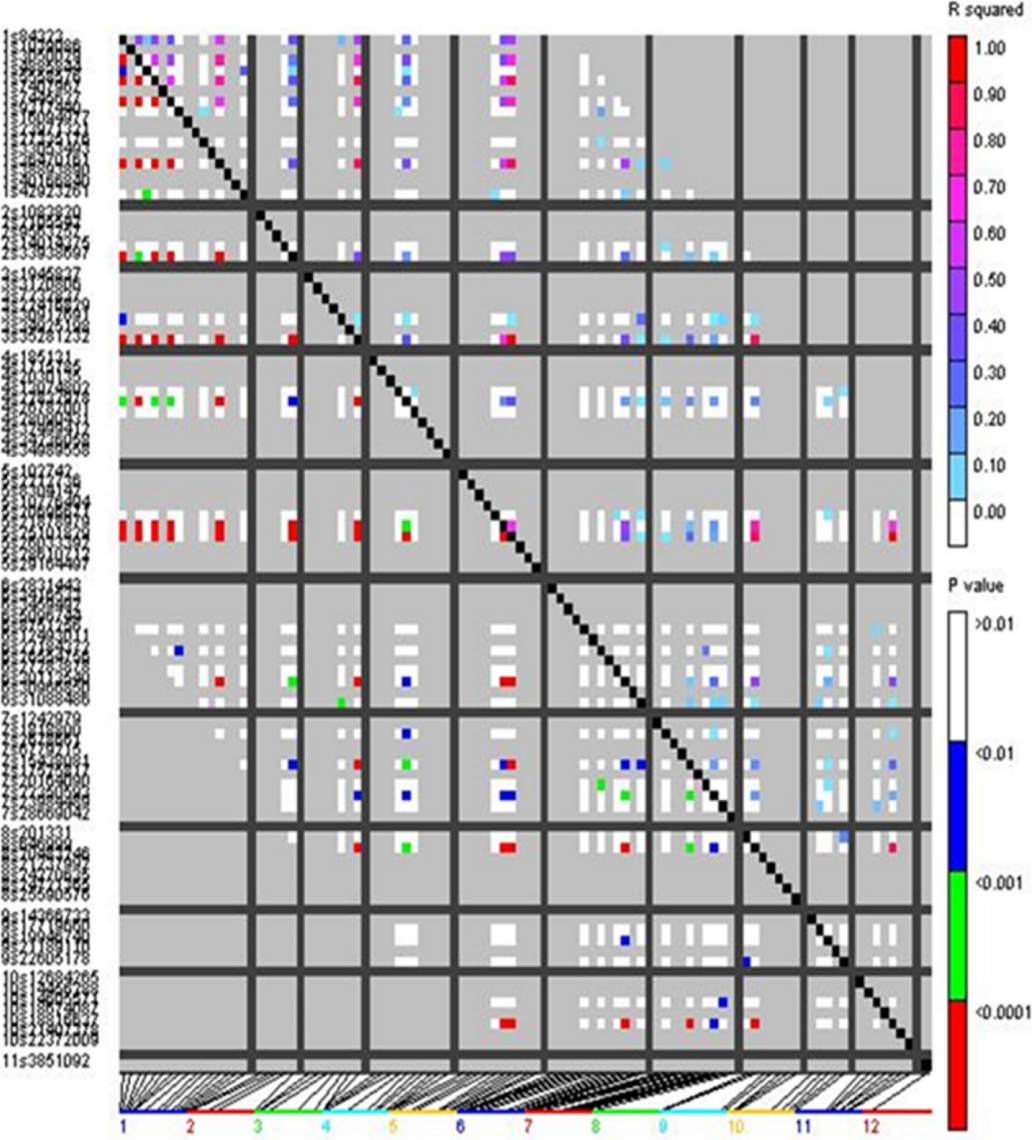
LD plot for a pair-wise SSR marker across rice genome used in genotyping diverse panel. Correlated markers were colour coded based on scales provided. Each pixel above the diagonal represents the *r*^2^ value and P-value of corresponding markers

The linkage disequilibrium (LD) map of the diverse panel (Panel I and II) revealed 8.25% of 100 SSR markers studied exhibited an R2 value of ≥0.1. In Panel I, the LD was unevenly distributed at genome level and concentrated on chromosomes 1 and 5. Among 52 LD hotspots identified, includes 46 inter-chromosomal LD pairs and 6 intra chromosomal LD pairs. LD plots with SSR markers demonstrated significant LD blocks are shown in Fig. 5. In Panel II, the LD was not uniform across the genome, higher LD values were observed on chromosomes 1, 2, 6, and 9 with 49 LD pairs spanning genomic regions.

### Mapping genomic regions with association analysis

The marker-trait associations for the phenotypic traits measured in diverse Panels I and II. In Panel I, the marker trait associations with GLM and MLM approaches identified 83 (Supplementary Table 5) and 16 (Table 4) significant loci, respectively. Especially the loci on chromosome 1, RM490 associated with CMS, CSI, RM259 associated with RWC, LR, LD, CMS, CSI, and SF identified with GLM approach, was identified by MLM approach. The loci, RM3825 on chromosome 1 associated with LT, RWC, CMS, CSI, Yield, 1000-GW, and SF (GLM) was detected in MLM approach with association with yield per plant.

**Table 4:** Marker trait association identified with MLM analysis in diverse panel I phenotypic data measured under water stress

Traits	Marker associated	Chromosome	***P*** value	***r***^***2***^ value
Leaf temperature (°C)	RM490	1	0.04996	0.05192
	RM259	1	0.02398	0.06945
Chlorophyll stability index (%)	RM490	1	0.00736	0.07886
	RM259	1	0.00775	0.07778
Yield per plant (g)	RM259	1	0.02936	0.06402
	RM3825	1	0.02105	0.07209
Thousand grain weight (g)	RM5961	11	0.0312	0.05483
Spikelet fertility (%)	RM259	1	0.0098	0.07782
	RM1031	6	0.04376	0.04657
Leaf rolling score	RM1026	9	0.00386	0.10547
Leaf drying score	RM259	1	0.03024	0.05067
	RM1026	9	0.01671	0.06224
Relative yield reduction (%)	RM5633	4	0.04017	0.05729
	RM1130	6	0.01309	0.08492
Drought susceptibility index	RM5633	4	0.04094	0.05682
	RM1130	6	0.01345	0.08419

In Panel II, the GLM approach detected 33 genomic regions associated with eight traits across genome except chromosomes 3 and 5 (Supplementary Table 6) with phenotypic variation ranging from 4.8 to 15.9%. The MLM approach detected 21 genomic regions (Table 5) associated with nine physiological and root architectural traits measured. Three new genomic regions that were not detected in GLM approach were identified with MLM approach. Three regions, RM283 (Chr.1), RM474 (Chr.10), RM5923 (Chr.11) were found only in MLM approach to be associated with SLA, SDW and RDW respectively. For example, the region RM1032 on chromosome 1 associated with plant height and root length, RM5961 on chromosome 11 was found to be associated with PH, CID and SLA.

**Table 5:** Marker trait association identified with MLM analysis in diverse panel II phenotypic data measured under water stress

Trait	Marker associated	Chromosome number	*p-v*alue	*r*^*2*^ value
Plant Height (cm)	RM1032	1	0.02369	0.06739
	RM5961	11	0.0272	0.06414
Root Length (cm)	RM1048	7	0.01914	0.07245
	RM1019	8	0.02792	0.06353
	RM1032	1	0.03786	0.05648
Root Volume (cm^3^)	RM5715	12	0.01405	0.07992
	RM1178	2	0.01452	0.07911
	RM246	1	0.03366	0.05918
Root dry weight (g)	RM5923	11	0.04139	0.05444
Shoot dry weight (g)	RM1178	2	0.00507	0.10531
	RM474	10	0.03569	0.05783
SCMR- Chlorophyll content (%)	RM5633	4	0.03022	0.06169
	RM1178	2	0.03806	0.05636
Chlorophyll content	RM1026	9	0.01678	0.07561
	RM259	1	0.03092	0.06115
	RM5633	4	0.04763	0.05126
Carbon isotope discrimination (Δ^13^C) ratio	RM5961	11	0.01652	0.07598
	RM1019	8	0.01918	0.07241
Specific leaf area (cm^2^)	RM5961	11	0.01369	0.08054
	RM5633	4	0.02136	0.06984
	RM283	1	0.04124	0.05453

### Co-location of QTLs with known drought related traits in rice

Especially the genomic regions detected in Panel II with majority of rice landraces on chromosome 1 (RM246), 7 (RM1048), 9 (RM1026), and 12 (RM5715) governing both physiological and root structure under water limitation are valuable genomic targets for rice trait breeding approach. The RM246 QTL was found to be linked with submergence [68], salinity tolerance [67], root biomass, basal root thickness [68] and yield [69, 70]. The RM1048 QTL was found to be linked with plant height [71], panicle length [72], leaf rolling [73] and cold tolerance [74]. The common regions, RM1026 and RM5961 detected in both panels co-locate QTLs for tiller number [66], biomass and grain yield [75], respectively.

### Tissue specific expression of genes underlying major QTL regions

Based on the physical position of markers associated with key traits, genes underlying QTLs identified were tabulated (Tables 6 and 7). In Panel I, eight loci on chromosomes 1, 4, 6, 9 and 11 flanking eleven genes were identified (Table 6). Among 11 genes, only 9 genes exhibited expression in Affymetrix datasets whereas all genes showed expression pattern in RNA-transcriptome. Among 13 genes identified in Panel II (Table 7), 10 genes exhibited tissue specific expression in rice grown in non-stress conditions.

**Table 6:** Genes underlying common genomic regions identified in marker trait association in diverse panel I

Marker	Chr.	Physical position (Mb) ^**a**^	Traits associated		Genes within confidence interval ^**a**^
			GLM	MLM	
RM259	1	7446642..7446813	CSI, SF, LD, LR, RWC	LT, CSI, SF, LD, Yield per plant	Phosphatidylinositol 3- and 4-kinase family protein LOC_Os01g13360
RM490	1	6677153..6677249	CSI, CMS	LT, CSI	peptidase C45 LOC_Os01g12230 Hypothetical protein LOC_Os01g12240
RM3825	1	36471204..36471354	LT, RWC, CMS, CSI, SF, 1000GW, Yield per plant	Yield per plant	Ras-related protein LOC_Os01g62950 Hypothetical protein LOC_Os01g62960
RM5633	4	13084092..13084302	PH, DFF	DSI, RYR	Retrotransposon protein LOC_Os04g23030 Expressed protein LOC_Os04g23040
RM1130	6	27284878..27285006	–	DSI, RYR	DNA binding protein LOC_Os06g45110
RM1031	6	31083106..31089543	CMS, RWC	SF	Expressed protein LOC_Os06g51330
RM1026	9	22605659..22605822	PH	LR, LD	CTR1-like protein kinase LOC_Os09g39320
RM5961	11	19926656..19926784	–	1000GW	Hypothetical protein LOC_Os11g34070

*Abbreviations*: *CMS* Chlorophyll Membrane Stability, *CSI* Chlorophyll Stability Index, *DSI* Drought susceptibility index, *LD* Leaf Drying, *LR* Leaf Rolling, *LT* Leaf Temperature, *RWC* Relative Water Content, *RYR* Relative Yield Ratio, *SF* Spikelet Fertility, *1000GW* Thousand grain weight

^a^ The physical position of SSR markers and genes underlying were obtained from www.ricebase.org

**Table 7:** List of genes underlying genomic regions identified in both GLM and MLM approaches with association analysis in diverse panel II

Marker	Chr.	Physical position (Mb) ^**a**^	Traits associated		Genes within confidence interval ^**a**^
			GLM	MLM	
RM259	1	7446642–7446813	Chl	Chl	Phosphatidylinositol 3- and 4-kinase family protein (LOC_Os01g13360)
RM1032	1	9318464–9318612	PH, RL	PH, RL	NB-ARC domain (LOC_Os01g16400) Actin (LOC_Os01g16414)
RM246	1	27336221–27336333	SPAD	RV	CS domain containing protein (LOC_ Os01g47770)
RM1178	2	14020245–14020356	RV, SDW, CT	RV, SDW, SPAD	Expressed protein (LOC_Os02g24205)
RM5633	4	13084092–13084302	RL, RV	SPAD, Chl, SLA	Retrotransposon protein (LOC_Os04g23030) Expressed protein (LOC_Os04g23040)
RM1048	7	20165312–20165449	RL, CT	RL	NB-ARC domain (LOC_Os07g33730
RM1019	8	202331–202477	RL, SPAD, Chl	RL, CID	Broad Complex BTB domain (LOC_Os08g01320)
RM1026	9	22605659–22605822	RL, RV, Chl	Chl	CTR1-like protein kinase (LOC_Os09g39320)
RM5961	11	19926656–19926784	Chl	PH, CID, SLA	Hypothetical protein (LOC_Os11g34070)
RM5715	12	25034080–25034280	RV, RL, Chl	RV	Biotin--protein ligase (LOC_Os12g40450) C3HC4 type domain protein (LOC_Os12g40460)

*Abbreviations*: *Chl* Chlorophyll content, *PH* Plant Height, *RL* Root length, *RV* Root Volume, *SPAD* Chlorophyll content (%), *SDW* Shoot Dry Weight, *CT* Canopy Temperature, *SLA* Specific Leaf Area, *CID* Carbon Isotope Discrimination

^a^ The physical position of SSR markers and genes underlying were obtained from www.ricebase.org

In Panel I, four genomic regions, RM490, RM3825 (chromosome 1), RM1130 and RM1031 (chromosome 6) were not detected in Panel II. The peptidase C45 gene (LOC_Os01g12230) associated with chlorophyll stability index was highly expressed in flag leaf in Affymetrix [76] and transcriptome study [77]. Between two loci underlying genomic region RM3825, the Ras-related protein (LOC_Os01g62950) exhibited higher expression in pollen [78] and anther [79]. The gene, DNA binding protein (LOC_Os06g45110) associated with RYR and DSI showed higher expression in root tip [80] specially in root elongation zone [81]. The expressed protein encoding gene, LOC_Os06g51330 exhibited higher expression of 14 fold in sperm cell [78] and callus [82].

Between Panel I and II, interestingly four locus (RM259, RM5633, RM1026 and RM5961) harboring five genes (LOC_Os01g13360, LOC_Os04g23030, LOC_Os04g23040, LOC_Os09g39320 and LOC_Os11g34070) were common and identified to be linked to key plant physiological production traits in rice. Gene expression analysis revealed the gene (LOC_Os01g09550) associated with SLA to be highly expressed in internode of rice dwarf mutant Fukei71 [83], treatment with gibberellin [84] and brassinosteroid [85]. The loci RM259 associated with chlorophyll content underlying gene, LOC_Os01g13360 was found to highly express in endosperm [86] and caryopsis [87]. The Loci, LOC_Os01g16400 associated with RL was found to highly expressed in root tip [88] and LOC_Os01g16414 associated with PH was highly expressed in shoot apex [87] and embryogenesis (PRJNA412710). The locus, LOC_Os04g23040 associated with SPAD, Chl, SLA on MLM approach was found to be highly expressed in flag leaves in both seedling and grain filling stages [77, 89] and leaf blade tissues [84, 90]. LOC_Os08g01320 associated with RL, SPAD, Chl (GLM approach) and RL, CID (MLM approach) was found to highly express in flag leaf [89], leaf blade (PRJNA392837) and root tissues (GSE24977). LOC_Os12g40460 associated with RV in both approaches were highly expressed in radicle tissue [91, 92].

Tissue specific expression data from global RNA transcriptome datasets revealed some genes not represented in Affymetrix arrays like, LOC_Os02g24205 associated with RV and SDW to be highly expressed in anther and pollen tissues [79], LOC_Os07g33730 associated with RL expressed in callus tissues [82]. The locus, LOC_Os09g39320 associated with chlorophyll content was found to highly express in flag leaf [77]. The genes, biotin--protein ligase (LOC_Os12g40450) and zinc finger, C3HC4 type domain protein (LOC_Os12g40460) associated with root volume was found to highly expressed in rice callus [82] and embryo [93].

### Genes underlying major QTL regions associated with drought stress

In Panel I, analysis of 11 genes with stress transcriptome datasets revealed six genes (LOC_Os01g12230, LOC_Os01g62950, LOC_Os04g23040, LOC_Os06g45110, LOC_Os06g51330, LOC_Os09g39320) to be highly expressed in rice tissues. The LOC_Os01g12230 (CSI), LOC_Os04g23040 (DSI, RYR) and LOC_Os06g51330 (SF) were highly expressed in leaf tissues under drought [94]. Intriguingly, the LOC_Os01g62950 (yield per plant), LOC_Os06g45110 (DSI and RYR) and LOC_Os09g39320 (LR, LD) were found to be highly expressed in 4 week old seedling root tissues under drought [95].

**Fig. 6:**
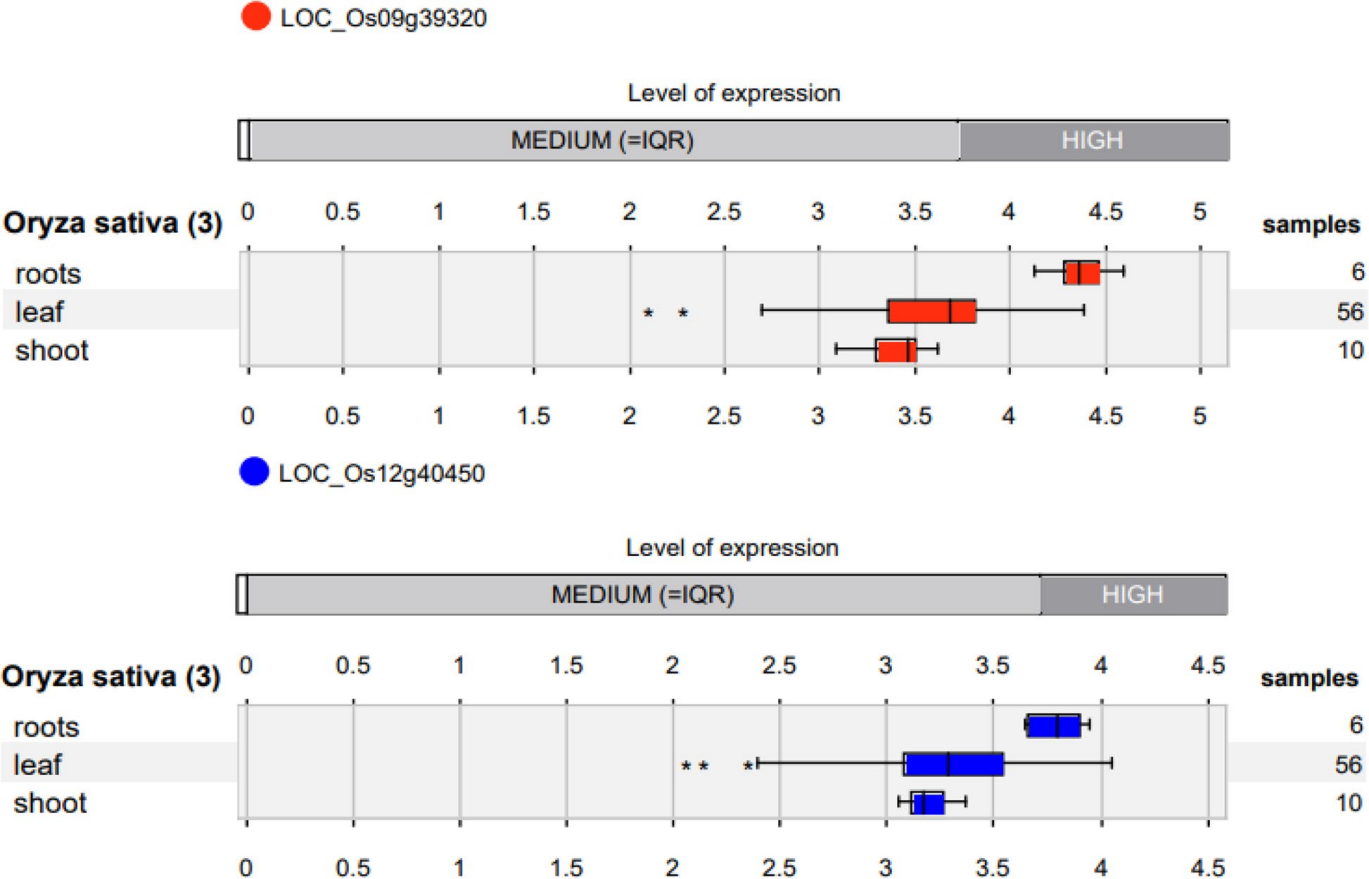
Expression profiling of candidate genes underlying root architectural and chlorophyll content on chromosome 9 and 12 identified in this study from drought stress RNA transcriptome data within Genevestigator software

In Panel II, evaluation of rice stress related gene expression datasets revealed LOC_Os01g09550 and LOC_Os01g16414 associated with SLA and PH to highly express in leaf tissue and panicle under stress [96]. These similar loci exhibited higher expression in rice root transcriptome study [95] and in leaf tissues under drought (PRJNA306542). The locus, LOC_Os04g23040 associated with different leaf traits was found to highly expressed in flag leaf [97] and other leaves under drought [94, 96, 98]. LOC_Os08g01320 associated with above and below ground root traits were found highly expressed in both flag leaf and root tissues under drought [94, 95, 97, 99]. LOC_Os12g40460 associated with RV in both mapping approaches, showed higher expression in root tissues under drought [100]. Drought stress specific global RNA transcriptome datasets revealed certain genes like LOC_Os02g24205 to be highly expressed in leaf in drought stress studies in rice [94, 98]. Whereas the locus, LOC_Os07g33730 associated with RL was found to be highly expressed in root tissues under drought [95]. The locus, LOC_Os09g39320 associated with root traits and chlorophyll content was found to highly expressed both roots and leaf tissues (Fig. 6) under drought [94, 95, 98]. Among two genes near the locus RM5715, biotin-protein ligase (LOC_Os12g40450) associated with root volume showed higher expression in root tissues (Fig. 6) under drought stress [95].

### Gene targets and SNP haplotype variation for trait introgression

Allele mining approach was employed with GBS data of selected 11 accessions (9 landraces [Chenkazhama; PTB26, Cholmala, Gandhakasala, Jeerakasala, Kalladiyar, Ponnaryan; PTB2, Thavalakannan; PTB8, Thekkan Cheera; PTB10 and Thonnuran] and 2 improved varieties [Athira; PTB51 and Jaya]) from a previous published literature [101]. Field trials in this study revealed these 9 landrace accessions to have desired phenotypic variances in Trials 1–4 than two improved varieties. Comparing four field trials, PTB2 recorded higher CSI (trials 1 and 2), PH, RL, RV, RB and Shoot biomass (trials 3 and 4). PTB8 was identified to be possess higher phenotypic variation for grain yield, CSI (trials 1 and 2), whereas for PH, CID, SLA and Root traits (RL, RV) in trials 3 and 4. PTB10 also recorded lower LR and LD, higher 1000GW, and yield, whereas this accession exhibited lower SLA, PH, root traits (RL, RV and RB) and shoot biomass in trials 3 and 4. PTB25 recorded lower LR and LD, DSI values, higher 1000 GW (trials 1 and 2) and higher shoot biomass (trials 3 and 4). In trials 1 and 2, PTB26 recorded higher SF and seed yield, whereas exhibited higher PH and shoot biomass in trials 3 and 4. Jeerakasala recorded lower RYR (trials 1 and 2) and higher PH (trials 3 and 4). Gandhakashala recorded lower DFF (trials 1 and 2) and CT than elite varieties, Jaya and PTB 51. Kalladiyar is an accession evaluated only in trials 3 and 4, found to have lower CID, lower SLA, higher RL and RV and shoot biomass.

Sequence level variation between *Oryza sativa* ssp. Indica reference genome to 93 local landraces from Kerala, revealed alternative haplotypes in key genomic regions identified in this study (Table 8). Further a deletion of sequence ‘ACCATCCATC’ (9318232 Mb) was found in all accessions evaluated in this study with single base pair, C in contrast to reference genome. Further sequence level variation was compared between two elite varieties used (PTB51 and Jaya) with nine landraces studied (Table 9). The genomic region, around RM1032 on chromosome associated with PH and RL, revealed two SNPs different in PTB8 (Thavalakannan) than elite varieties. PTB8 (Thavalakannan) and Kalladiyar accessions also recorded lower SLA were found to have three different SNPs around RM5633 region (Table 9). PTB26 also found to harbor novel allele to improve SF (31089179 Mb) on chromosome 6 near RM1031 under water stress. Mining another genomic region on chromosome 11 associated with 1000GW revealed the accession PTB25 (Thonnuran) to have allele similar to elite variety, PTB51, whereas other accessions PTB26 (Chenkayama) and Gandhakasala have novel alleles than elites for use as future breeding targets.

**Table 8:** List of haplotype variation identified in genomic regions with sequence information on selected rice landraces

Chr. (Physical position in Mb^**a**^)	Marker located	Reference Allele	Alt Allele	Position (Mb)	SNP type	Traits associated in MLM approach
CHR1 (9318264–9318812)	RM1032	T C	C A	9318278 9318295	Up-stream gene variant Intergenic region variant	PH, RL (Panel II)
CHR4 (13083892–13084502)	RM5633	T A A	C T C	13083944 13083963 13084032	Intergenic region variant Intergenic region variant Intergenic region variant	DSI, RYR (Panel I) SPAD, Chl, SLA (Panel II)
CHR6 (31082906–31089743)	RM1031	G	A	31089179	Down-stream gene variant	SF (Panel I)
CHR11 (19926456–19926984)	RM5961	T A	G G	19926506 19926513	Down-stream gene variant Down-stream gene variant	1000GW (Panel I) PH, CID, SLA (Panel II)

*Abbreviations*: *Chl* Chlorophyll content, *CID* Carbon Isotope Discrimination, *DSI* Drought susceptibility index, *PH* Plant Height, *RL* Root length, *RYR* Relative Yield Ratio, *SPAD* Chlorophyll content (%), *SLA* Specific Leaf Area, *SF* Spikelet Fertility, *1000GW* Thousand grain weight

^a^ The physical position of SSR markers and genes underlying were obtained from www.ricebase.org

**Table 9:** List of SNP variation identified in rice landraces for potential use as molecular markers in drought rice breeding

Trait Association (Marker linked)	Chr. Phys. position (Mb) ^**a**^	Elite allele	Position (Mb)	Landrace allele	Landrace allele present in
PH/RL (RM1032)	CHR1 9318264–9318812	C A	9318278 9318295	T C	PTB 8
SPAD/ Chl/ SLA (RM5633)	CHR4 13083892–13084502	C T C	13083944 13083963 13084032	N N N	All four accessions: PTB8, Kalladiyar, Gandhakashala, Jeerakashala
PH/CID/SLA/1000GW (RM5961)	CHR11 19926456–1926984	T	19926506	N K G	PTB51, PTB25 Gandhakashala PTB26
		A	19926513	N R G	PTB51, PTB25 Gandhakashala PTB26
SF RM1031	CHR6 31082906–31089743	R	31089179	A G R	Kalladiyar PTB2, 10, 25 and 26, Gandhakashala, Jeerakashala PTB8, Chomala

*Abbreviations*: *Chl* Chlorophyll content, *CID* Carbon Isotope Discrimination, *PH* Plant Height, *RL* Root length, *SPAD* Chlorophyll content (%), *SLA* Specific Leaf Area, *SF* Spikelet Fertility, *1000GW* Thousand grain weight

^a^ The physical position of SSR markers and genes underlying were obtained from www.ricebase.org

## Discussion and conclusion

Rice is a staple food for more than half of global population and South East Asia as a origin of rice with 88,681 different varieties which includes 55,615 landraces [102]. Rice land races are valuable genetic resources for improving agronomic traits, resilience and yield optimization under stress environments [60, 61]. Rice cultivation in Kerala, south-western corner of the Indian peninsular region dates back to 3000 B. C, holds valuable genetically diverse wild and cultivated rice accessions [103]. Recent efforts in rice breeding program enabled genetic improvement towards drought adaptation through conventional mapping approach [12, 14, 104, 105], marker assisted selection of high yielding varieties or by selection of secondary traits related to drought tolerance [10, 13, 23, 106]. Wild crop relatives, wild/weedy species and primitive landraces are valuable genomic resources to identify new alleles for yield improvement in domesticated crop species [107] are yet to be explored. Recently wild soybean was found to harbor novel alleles to enhance root system architecture and seed yield in cultivated soybean genetic backgrounds [34, 108–110].

To our knowledge this is the first study on 99 diverse accessions specific to Kerala State of India, characterized with SSR markers, which are highly evolutionally neutral spanning complete rice genome. Among the classical markers, SSRs were selected based on several desirable genetic attributes like reproducibility, multi-allelic, wide distribution in both coding and non-coding regions of the genome. Moreover association analysis with rice accessions were successfully conducted with number of genotypes, less than or little over to 100 genotypes to identify genomic regions associated with different polygenic traits in rice. For example, Shomura et al. [111] mapped major loci governing grain length, grain width, grain width-length ratio using 84 rice landraces. Thurber et al. [112] studied 105 weedy rice accessions to map shattering loci to understand the influence of evolutionary pathway on rice domestication. In addition to the difficulty involved in root phenotyping approaches, researchers were successful in identifying genes associated with various root architectural traits in rice with 93 temperate [113] and field study with 49 [114] landrace accessions, in wheat which is very complex in contrast to rice genome composition and ploidy levels by using 91 genotypes [115] and with model plant, *Arabidopsis* (96 accessions) grown under nitrogen [116] and potassium nitrate [117] treatments respectively .

Our study with two diverse rice panels, reveled valuable genetic resources to improve physiological processes and productivity in rainfed ecosystem. Combined phenotypic data across trials and panels revealed certain accessions as valuable genetic resources to increase shoot biomass [PTB10, PTB26], lower drought susceptibility index [PTB 25, Bhadra], and higher grain yield and spikelet fertility [PTB1, PTB60]. Certain accessions like PTB1, 2, 10, 13, 15, 29, 30 were found as genetic resources to stack different traits to improve rice plant performance and yielding ability under drought with optimized root system. GWAS analysis revealed rice genomic region to be associated with phenological, physiological and root traits measured across seasons in this study. GWA studies in rice were successful in identifying genomic regions for agronomic [118] and root traits [47, 65, 119].

Comparing two diverse panels, uncommon genomic targets between panels, Panel I (RM1031) and Panel II (RM0132 and RM5633) were selected. Mining of publically available transcriptome datasets reveled the gene, LOC_Os06g51330 underlying RM1031 region associated with SF will be an interesting target to increase seed setting under stress. The genes underlying Panel II specific targets (RM0132 and RM5633), LOC_Os01g16414 and LOC_Os04g23040 associated with plant height, leaf physiological and root architectural traits are valuable targets to improve rice productivity under stress. Co-location of genomic regions identified in this study with previous rice QTL/association mapping studies [66–75, 120] are encouraging and are key candidate targets for introgression to develop next generation rice varieties drought tolerance. Seed yield under stress is a complex trait and a seamless benefit occurs out of coordinated activity of root and shoot physiological traits combined. Thus, identifying genomic regions that are associated with key physiological parameters and root traits are critical to optimize soil resources and improve yield under stress. The accessions, PTB2 (Ponnaryan), PTB8 (Thavalakannan), PTB10 (Thekkancheera), PTB25 (Thonnuran), PTB26 (Chenkayama), PTB30 (Chuvanna Modan), Gandhakashala, Jeerakasala and Kalladiyar are valuable genetic resources to combine different physiological, root architectural and seed yield to improve stress resilience and productivity in rainfed ecosystem. Key genomic targets identified on chromosome 1 (PH/RL), 4 (chlorophyll content/SLA), 6 (SF) and 11 (PH/CID/SLA/1000GW) and their allelic variation in the genes underlying these QTLs are novel targets for use in rice drought breeding program. The SNPs identified in these key genomic targets are valuable and have potential to be developed as KASP markers to facilitate tracking and introgression of these target traits into elite rice cultivars suited to rainfed ecosystem.

## Methods

### Field evaluation of diverse panel for physiological and agronomic traits (trials 1 and 2)

#### Genetic materials

Eighty-one diverse rice accessions (Panel 1) used for drought screening and identify marker trait association are listed in Supplementary Table 1. This panel includes indigenous land races (45 accessions) and improved cultivar (36 varieties) from R.A.R.S., Pattambi, Kerala Agricultural University and National Rice Research Institute, Cuttack (ICAR-NRRI), Odisha. Most of the landraces studied had an average yield potential of 2-3 t/ha, tolerant to major pests and water stress. The improved varieties were short and medium duration with red or white bold grain type with yield potential of 5-6 t/ha, but with moderately tolerant or susceptible to abiotic stresses. This study protocol comply with relevant institutional, national, and international guidelines and legislation.

#### Experimental details

The experiment was conducted at Regional Agricultural Research Station, Pattambi, Kerala Agricultural University, India (10°48′41.1″N 76°11′24.9″E), oldest rice research station in Kerala with managed irrigation facility for drought stress phenotyping studies. This experimental site is rice target environment and severely prone to heat and drought stresses. The selected diverse panel were grown in field site during wet season; Mundakan (August –December) of 2017 (Trial 1) and dry season; Puncha (January –May), 2018 (Trial 2). The experiment was laid out in alpha lattice design with two replications in both control and irrigated treatments. In both seasons, wet (Trial 1) and dry (Trial 2), 10 g seeds of each genotype were planted in pots filled with soil, sand and cattle manure with equal v/v ratio. Twenty-one days old seedlings were transplanted to the open field at the rate of 2 seedlings/ hill. After 8 days of transplanting, gap filling was done and one healthy seedling was maintained per hill. Each genotype was transplanted in four rows of 2 m length with a spacing of 20x15cm. Management practices were followed as per package of practices recommendation of Kerala Agricultural University. Fertilizer dose of N: P: K in the ratio of 90:45:45 Kg/ha in three split doses @ 1/3 dose of each fertilizer at basal, tillering and at panicle initiation stages. Blast incidence was controlled by using Nativo @ 4 g/10 l of water to impose water stress, irrigation was withheld for 25 consecutive days at the panicle initiation stage. Measurements on morphological and physiological parameters were recorded after 10 days of stress imposition. After stress period, re-watering was done at reproductive stage and maintained till physiological maturity. During harvest, data on plant production traits were recorded in both control and drought treatments. To quantify the soil moisture and the level of water stress data on moisture content was estimated using gravimetric method.

Physiological parameters such as leaf rolling score and drying score was done according to the Standard Evaluation System for Rice (SES) of IRRI, Philippines. Leaf rolling and drying scores were taken after 10 days of drought imposition as when leaf tissues showed drought symptoms during the time between 12 pm and 1 pm. Leaf temperature was measured using infrared thermometer (AG-42, Teletemp Corporation, CA, USA) with an 8° field of view and equipped with a 10.5- to 12.5-μm band pass filter, as described [121]. The measurement was made at noon by facing south to minimize the effects of sunlight. Cell membrane stability index was estimated as per the procedure described [122]. The relative leaf water content in percentage was measured based on the method described [123]. Total chlorophyll content was estimated by the method [124] and from that chlorophyll stability index was calculated using formula. Chlorophyll stability index (%) = (Total chlorophyll content at drought stress/ Total chlorophyll content at irrigated condition) X 100. Plant production traits like yield per plant was derived weighing filled grains in each panicle expressed in grams. Thousand-grain weight was measured from seeds selected randomly from each replication and weighed. Spikelet fertility (%) was calculated as per standard formula, Spikelet fertility (%) = (Number of fertile spikelet/ Total number of spikelets) X 100. Drought susceptibility index was measured according to Fisher and Maurer [125]. Percentage relative yield reduction (RYR) under stress was computed as per following equation: RYR = 1 – (grain yield in drought stress / grain yield in irrigated condition) X 100.

### Evaluation of root architectural traits in diverse rice panel study (trials 3 and 4)

A previous study [25] with another diverse panel of 81 accessions (Panel II) adapted to target rainfed environments (Supplementary Table 1). This panel includes medium duration rice landraces and improved varieties with average yielding potential of 2.5 t/ha (45) and 4–5 t/ha (36), respectively. Only 63 rice accessions are common across Panel I and II studied in different rice seasons prevalent in rice ecosystem of Kerala at the field with root phenotyping facility located. Rice seeds were planted in a specially constructed “root structure” of 5 ft. × 10 ft. × 60 ft. (H x W x L) during cropping season, Virippu (June –September) in 2011and 2013. Five seeds per accessions were sowed in “root structure” which is located at 10°48′41.1″N 76°11′24.9″E within Regional Agricultural Research station (RARS), Pattambi, Kerala Agricultural University (KAU), India. Thinning was done on 18th day and retained only one plant/hill. Each genotype were planted in a row of 2 m length with a spacing of 20 × 15 cm and replicated twice. The crop was sole depend on rainfall as source of irrigation. Fertilizer dose of N:P:K in the ratio of 90:45:45 Kg/ha in three split doses @ 1/3 dose of each fertilizer at basal, tillering and at panicle initiation stages. Blast incidence was controlled by using Nativo@4 g/10 L water. After 60 days after planting (DAP) measurements on Specific leaf area (SLA) and SPAD measurement on chlorophyll content was made as detailed [25]. Seventy days after sowing root architectural traits were measured.

Five leaves from actively growing rice seedlings were collected for CID analysis 50 days after sowing. Fully opened leaves were dried in oven at 80 °C for 3 days and powered the leaves using ball and mill. The water use efficiencies (WUE) of 81 rice accessions studied in the study was measured using ∆^13^C values, negatively associated with WUE. An isotope ratio mass spectrometer (IRMS) interfaced with a suitable combustion system is used for the determination of ∆^13^C. The plants ability to discriminate against the heavy isotope of carbon (^13^C), resulting in the depletion of 13C content in biomass compared with atmospheric air during photosynthesis activity was used to measure CID [126]. Δ^13^C was measured as the ratio of the partial pressures of CO_2_ inside the leaf to that in ambient air (Pi/Pa), as follows: Δ^13^C = a + (b − a) Pi/Pa; where, a and b are isotope fractionations that occur during diffusion through stomata and carboxylation by Rubisco respectively.

SPAD (SPAD 502 plus chlorophyll meter, Spectrum Technologies, Inc.) was used to measure the chlorophyll content based on light absorbance and/or transmittance characteristics at 430 nm and 750 nm on a leaf tissue. Percent chlorophyll content was measured between 10.00 and 16.00 h on second or third fully expanded leaf lamina avoiding the mid-rib portion. Actual chlorophyll content was measured using lab based assay [124]. The specific leaf area (SLA) is measured as the ratio of leaf area to leaf dry weight, an indirect measure of leaf expansion as described [25]. Higher SLA phenotypic values represents larger leaf surface area available for transpiration, inversely related to WUE. Data on SLA was measured on completely expanded second or third leaf from main stem apex using a leaf area meter (CI-203, CID Bioscience, UK). Dry weight on the leaf used to measure SLA was collected after dried in a hot-air oven at 70 °C for 3 days and weighed. SLA was calculated as using the formula as below: SLA (cm-2 g-1) = (leaf area / leaf dry weight).

After 70 days of planting data on plant height and biomass were collected. Then the brick wall in the root phenotyping structure was dismantled and strong jet of water was used to remove the excess soil adhering plant root system. The soil particles adhering close to rice roots were removed carefully and data on root length, root volume, root dry weight were measured as described [25]. Root length was manually measured using a ruler from shoot tiller-root junction to the deepest root. Root volume was measured in cubic centimeter by water displacement method, by immersing cleaned soil free roots into a measuring cylinder with 1 l of water. Root dry weight was obtained from oven dried roots at 80 °C for 48 h.

### Effect of reproductive stress on rice agronomic traits (trial 5)

A subset of 35 diverse lines (Supplementary Table 1) within 81 diverse panels was evaluated under drought stress imposed on reproductive stage stress following a Completely Randomized Design in a rainout shelter of Department of Plant Physiology, College of Agriculture, Kerala Agricultural University during the year 2017. The subset genotypes were raised in polythene tubes of 25 cm diameter and 1 m height. Plants in both control and drought treatments were irrigated regularly until panicle initiation (PI) stage. After PI, irrigation was withheld for a period of 15 days in drought treatment PVCs to evaluate leaf, root and yield responses under reproductive stage water limitation. The control plants are irrigated regularly till maturity. The methodology used to quantify leaf-rolling response and root traits are published earlier [26].

### Molecular marker genotyping

Leaf tissues of 20 days old plants on 81 diverse rice panel were collected in a small Ziploc bags in an ice cooler to extract genomic DNA. The leaf samples were stored at − 80 °C until processed. Genomic DNA was extracted using the method by Dellaporta et al. [127] and the quality of resultant DNA was determined using spectrophotometer (ELICO, SL 21 UV-Vis spectrophotometer) based on absorbance ratio of 260 nm to 280 nm (A 260/A 280) between 1.7 and 1.8 [128]. DNA quality was also assessed using gel electrophoresis with 5 μl of crude DNA sample on agarose gel (0.8%) stained with ethidium bromide. A total of 100 SSR markers spanning whole rice genome across 12 chromosomes were selected from published rice literatures on drought tolerance (Supplementary Table 7). The genotyping was performed at College of Agricultural Vellayani, Thiruvannathapuram, Kerala Agricultural University (KAU), India.

Gradient cycle was performed in Master Cycler gradient 5331 (Eppendorf version 2.30.31–09, Germany) to optimize the annealing temperature suitable for SSR genotyping in rice diverse panel. A final sample volume of 20 μl was used for PCR reaction with reaction mixture contained 30 ng/μl DNA, 2.5 mM dNTPs, 30 ng forward and reverse primer each, 1 unit of Taq DNA polymerase with 10X reaction buffer and 25 mM MgCl_2_. Genotyping was performed using the following PCR cycle: 94 °C for 5 min (1x), 35 cycles each of 94 °C for 30 s, 50–60 °C (vary depend on SSR annealing temperature) for 30 s, 72 °C for 1 min with final cycle at 72 °C for 5 min and 4 °C for infinity. After PCR amplification, 3 μL of gel loading dye (Bromophenol blue) was added directly to the reaction tubes and spun for few seconds in a micro centrifuge. The PCR amplicons were resolved on 2% agarose gel stained with ethidium bromide along with marker (100 bp ladder). Electrophoresis (Bio-Rad, Deutsch) was performed using 1X TBE as buffer solution at 80 V with running time of 90 min. The gel was visualized under UV (312 nm) transilluminator in gel documentation system (Syngene G-box documentation system) and scored as 1 for presence or 0 for absence of specific allele.

### Association mapping analysis

Population structure of 81 rice genotypes was estimated using a STRUCTURE software V2.3.4 based on Bayesian clustering algorithm [128]. Five independent runs were made to detect the optimum number of subpopulations, with K values (K = 2 to 8) using burn and run lengths of 50,000. The results were imported to STRUCTUREHARVESTER software to calculate exact value of 1 K [129]. TASSEL v.5 software package was used to calculate Linkage Disequilibrium (LD) for 81 diverse rice accessions at *p*-value < 0.05. The analysis was conducted by considering genotypes with and without admixture identified by software STRUCTURE at K = 2. The LD was estimated by considering the squared correlation coefficient (r^2^) between SSR markers and plotted as triangle plots to evaluate LD blocks in association genetic analysis. The marker-trait association was calculated using TASSEL 5 based on General Linear Model (GLM) based on Q-matrix based on the population structure and a Mixed Linear Model based on Q-matrix and the kinship-matrix (MLM) [130]. In both models the markers with *p*-value < 0.05 and r^2^ value > 0.1 were considered as significant markers for further analysis. To identify genes underlying major QTLs flanked by SSR markers, two-hundred Kb (both up and downstream) around the SSR marker physical positions were mined using Ricebas e[131] and QTL co-location across published literature were obtained from Rice SNP-Seek Database [132]. In general, the average range of LD decay in different rice sub-populations ranged from 100 to 500 kb [133], thus two-hundred Kb (both up and downstream) around the SSR marker physical position were mined to identify possible causal loci underlying trait of interest. Similar approach of using SSR physical position to find causal loci were proven for various agronomic traits governed by polygenes like grain number [133] and drought tolerance [106], yield under drought [134] and nitrogen starvation [135]. The tissue specific expression pattern of genes underlying QTLs were mined within rice Affymetrix and global transcriptome data integrated into Genevestigator software [136].

### Genotyping by sequencing (GBS) of land races and elite varieties

GBS data on eleven accessions including 9 landraces (Supplementary Table 8) ([Chenkazhama; PTB26, Cholmala, Gandhakasala, Jeerakasala, Kalladiyar, Ponnaryan; PTB2, Thavalakannan; PTB8, Thekkan Cheera; PTB10 and Thonnuran]) and two elite varieties [PTB51 (Athira) and Jaya] were used for further analysis [101]. These eleven accessions represent wide variety of rice taxa [137] [PTB51 (Athira), PTB26 (Chenkayama), Chomala, Gandhakasala, Jaya, Jeerakasala, Kalladiyar, PTB2 (Ponnaryan), PTB8 (Thavalakannan), PTB10 (Thekkancheera) and PTB25 (Thonnuran)]. Accessions representing *Oryza meyeriana* subsp. Granulate from Wynad district: Chomala, Gandhakasala and PTB25 (Thonnuran) and PTB8 (Thavalakannan) from Kasaragod representing *O. sativa* f. spontanea (weedy rice) represents novel alleles that would improve grain yield and stress resilience in elite rice cultivars [137]. Each accession library was prepared after restriction digestion of 10–20 μg of genomic DNA by ApeK1 + Pst1 enzyme followed by ligation of barcoded adapters. Library quality check was performed using Agilent Tape Station and sequenced on Illumina Nextseq 500 platform with 2 × 150 bp v2 chemistry. Further details on read depth and SNP calling can be obtained from previously published literature [101].

### Identification of conserved SNP haplotypes among rice landraces

Whole-genome scans were performed by comparing GBS data between 2 elite rice lines and 9 landraces at nucleotide level to identify alleles similar across rice landraces but different than elite varieties. Each candidate gene underlying marker-trait association were analyzed to identify conserved genic single nucleotide polymophic (SNP) variation among rice landraces for potential use as marker to introgress drought tolerance in future rice breeding program. Similar approach for comparing deep sequence data on elite and landraces/wild accessions revealed a SNP allele with higher frequency among upland rice variety to regulate lateral root density [138].

### Study protocol comply with relevant institutional, national, and international guidelines and legislation

#### Statistical analysis

The phenotyping data were analyzed statistically using the SAS program (SAS institute Inc., 1990). Principal component analysis, principal co-ordinate analysis and cluster analysis were done using R environment of statistical computing (R core Team, 2013). Predictors for leaf rolling and grain yield under stress were identified using bootstrap forest model integrated into JMP SAS software (v.15.1.0).

#### Permission to collect samples

Memorandum of Understanding between institutions are needed to collect the samples from Regional Agricultural Research Station, Pattambi, Kerala, India.

## Supplementary Information

**Additional file 1**: **Supplementary Table 1**. List of rice diverse panels (Panel I and II) evaluated in the study.

**Additional file 2**: **Supplementary Table 2**. Correlation of phenological, physiological traits with yield under water stress in trial 1.

**Additional file 3**: **Supplementary Table 3**. Correlation of phenological, physiological traits with yield under water stress in trial 2.

**Additional file 4**: **Supplementary Table 4**. Correlation among morpho-physiological and root traits measured under rainfed condition in trial 3.

**Additional file 5**: **Supplementary Table 5**. Marker-trait association identified with GLM analysis in diverse panel I phenotypic data measured under water stress.

**Additional file 6**: **Supplementary Table 6**. Marker-trait association identified with GLM analysis in diverse panel II phenotypic data measured under water stress.

**Additional file 7**: **Supplementary Table 7**. List of SSR markers used in genotyping diverse rice panels (Panel I and II).

**Additional file 8**: **Supplementary Figure 1**. Variation in precipitation and relative humidity experienced by diverse rice panels evaluated in field trials 1–4. Trial 1(2017), Trial 2(2018), Trial 3(2011) and Trial 4 (2013).

**Additional file 9**: **Supplementary Table 8**. SNP variation identified in 11 rice land race accessions used in this study.

## Data Availability

Genotyping By Sequencing data with SNP information is available as Additional file 4. This SNP data can be shared on the request and mutual consent. Seeds of germplasm material are available from Regional Agricultural Research Station, Pattambi, Kerala Agricultural University, India. Please contact Dr. Manickavelu Alugu (amanicks@cukerala.ac.in) for the SNP data and Dr. Beena (beena.r@kau.in) for germplasm request.

## Abbreviations

GBS: Genotyping By Sequencing
WUE: Water Use Efficiency
QTL: Quantitative Trait Loci
GWAS: Genome Wide Association Study
DSI: Drought Susceptibility Index
CID: Carbon Isotope Discrimination
SLA: Specific Leaf Area
PCA: Principal Component Analysis
PC: Principal Component
PH: Plant Height
DFF: Days to 50% flowering
SB: Shoot Biomass
RB: Root Biomass
RL: Root Length
RV: Root Volume
SDW: Shoot Dry Weight
RDW: Root Dry Weight
SPAD: Soil Plant Analysis Department
RWC: Relative Water Content
CMS: Cell Membrane Stability
CSI: Chlorophyll Stability Index
SF: Spikelet Fertility
LT: Leaf Temperature
LR: Leaf Rolling
RYR: Relative Yield Reduction
LD: Leaf Drying
SSR: Simple Sequence Repeat
GLM: General Linear Model
MLM: Mixed Linear Model
DNA: Deoxyribo Nucleic Acid
RNA: Ribo Nucleic Acid
PCR: Polymerase Chain Reaction
PI: Panicle Initiation
