# Supplementary material for: Association mapping of drought tolerance and agronomic traits in rice (Oryza sativa L.) landraces
Source: BMC Plant Biol. 2021 Oct 23;21:484. doi: 10.1186/s12870-021-03272-3 (PMC8539776; doi:10.1186/s12870-021-03272-3)
Supplement: Supplementary file 8 — Additional file 8: Supplementary Figure 1. Variation in precipitation and relative humidity experienced by diverse rice panels evaluated in field trials 1–4. Trial 1(2017), Trial 2(2018), Trial 3(2011) and Trial 4 (2013). [file 12870_2021_3272_MOESM8_ESM.ppt]

## Slide 1
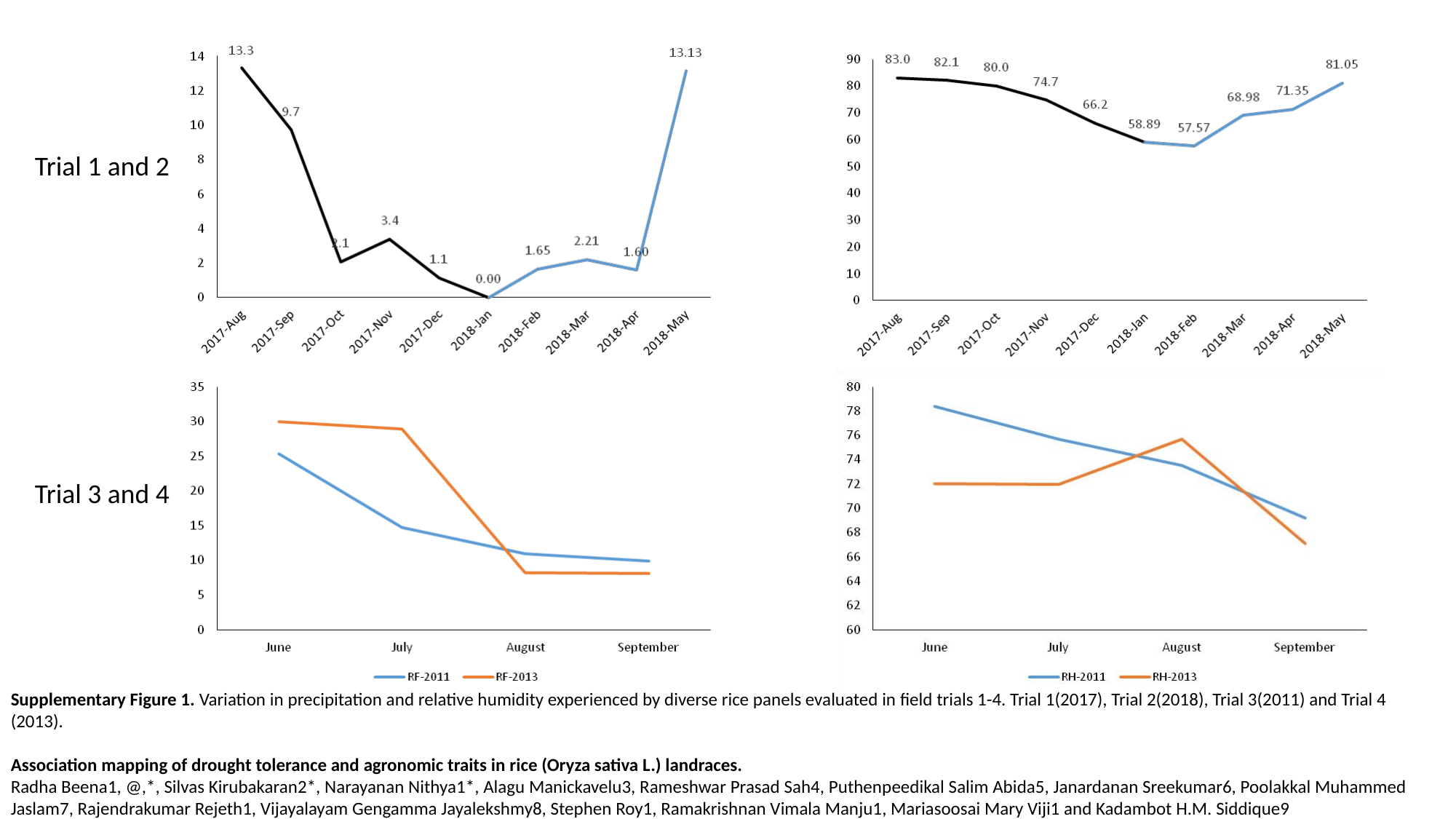

Trial 1 and 2
Trial 3 and 4
Supplementary Figure 1. Variation in precipitation and relative humidity experienced by diverse rice panels evaluated in field trials 1-4. Trial 1(2017), Trial 2(2018), Trial 3(2011) and Trial 4 (2013).
Association mapping of drought tolerance and agronomic traits in rice (Oryza sativa L.) landraces.
Radha Beena1, @,*, Silvas Kirubakaran2*, Narayanan Nithya1*, Alagu Manickavelu3, Rameshwar Prasad Sah4, Puthenpeedikal Salim Abida5, Janardanan Sreekumar6, Poolakkal Muhammed
Jaslam7, Rajendrakumar Rejeth1, Vijayalayam Gengamma Jayalekshmy8, Stephen Roy1, Ramakrishnan Vimala Manju1, Mariasoosai Mary Viji1 and Kadambot H.M. Siddique9
